# Association between composite dietary antioxidant index and increased urinary albumin excretion: a population-based study

**DOI:** 10.3389/fnut.2025.1552889

**Published:** 2025-03-28

**Authors:** Shaopeng Li, Suqiong Yang, Yong Wang, Zongqi Lin, Fuyuan Chen, Qinghe Gao, Jiantong Cai

**Affiliations:** ^1^Department of Urology, Shishi General Hospital, Quanzhou, China; ^2^Department of Psychiatry, The Third Hospital of Quanzhou, Quanzhou, China; ^3^Child and Adolescent Psychiatry, The Third Hospital of Quanzhou, Quanzhou, Fujian, China

**Keywords:** Composite Dietary Antioxidant Index (CDAI), Albumin-Creatinine Ratio (ACR), cross-sectional survey, cardiovascular disease, chronic kidney disease

## Abstract

**Introduction:**

The Albumin-Creatinine Ratio (ACR) is a key biomarker for early kidney disease detection and is predictive of chronic kidney disease (CKD) progression and associated cardiovascular risks. Antioxidant diets, indicated by the Composite Dietary Antioxidant Index (CDAI), may reduce oxidative stress and alter albumin urinary excretion rates. This study explores the relationship between CDAI and albuminuria.

**Materials and methods:**

Data on intake of vitamins A, C, E, zinc, selenium, and beta-carotene from the NHANES database (2007–2018) were used to compute CDAI scores. To measure urinary albumin, the ACR levels were assessed. The association between CDAI and ACR was analyzed through multivariate logistic regression, subgroup analysis, and interaction tests, incorporating a generalized additive model (GAM) to evaluate potential non-linear relationships.

**Results:**

A total of 28,601 participants were included with an average CDAI of 0.302 ± 3.895. Those in higher CDAI quartiles showed a reduced likelihood of elevated ACR. The prevalence of increased ACR decreased across the CDAI quartiles from 13.89% in Q1 to 10.11% in Q4. Higher CDAI scores were inversely related to ACR (OR: 0.99, 95% CI: 0.97–1.00), with a significant interaction effect by BMI (*p* = 0.0048). In males, a distinct L-shaped relationship was noted, with a negative correlation between CDAI and ACR to the left of the inflection point at 0.53 (OR: 0.95, 95% CI: 0.91–0.98).

**Conclusion:**

Increasing CDAI is associated with lower ACR and reduced risk of albuminuria, suggesting that dietary antioxidants may benefit renal and cardiovascular health.

## Introduction

The albumin-to-creatinine ratio (ACR), a novel composite indicator based on urinary albumin and creatinine measurements, has recently gained considerable attention. The ACR is calculated by dividing the urinary albumin concentration by urinary creatinine concentration, with an increased urinary albumin secretion defined as ACR > 30 mg/g (1). Elevated ACR has been identified as a predictor of early chronic kidney disease (CKD) in several studies (2–4). Most research has focused on the relationship between increased ACR and CKD, as well as on how independent risk factors predict elevated ACR (5, 6). However, recent studies have highlighted that microalbuminuria is not only a precursor to renal disease but also an independent predictor of cardiovascular events, especially in patients with diabetes or hypertension (7, 8). When the endothelium of blood vessels is damaged, their permeability increases, causing large molecules such as proteins to leak into the urine to form proteinuria. This association suggests that albuminuria may reflect systemic endothelial dysfunction, a critical mechanism in the progression of cardiovascular disease (9). Damage to the vascular endothelium is followed by secondary injury that promotes thrombosis, triggers an inflammatory response and leads to vasoconstriction, which exacerbates the progression of cardiovascular diseases such as atherosclerosis and hypertension, and increases the burden on the kidneys. Hence, monitoring the ACR is essential not only for diagnosing and tracking the progression of CKD but also for preventing and managing related cardiovascular diseases.

Overproduction of reactive oxygen species (ROS) is associated with the development of various chronic diseases such as cancer, neurodegenerative disorders, and cardiovascular diseases. Under normal physiological conditions, antioxidants regulate ROS levels and maintain cellular homeostasis. However, antioxidant deficiency can exacerbate oxidative stress and increase the risk of disease. The Composite Dietary Antioxidant Index (CDAI) is a scoring system that assesses an individual’s total antioxidant capacity based on dietary antioxidants, including vitamins A, C, and E, and minerals such as selenium and zinc. Existing literature indicates that a higher CDAI may reduce the risk of metabolic disorders such as hyperlipidemia, hyperuricemia, and osteoporosis. Additionally, a cross-sectional study involving 6,874 participants demonstrated the benefits of adequate dietary antioxidant intake for populations at a high risk of CKD. Nevertheless, the specific relationship between CDAI and the ACR remains unclear.

This study is the first to investigate the relationship between CDAI and ACR through a cross-sectional survey of the US National Health and Nutrition Examination Survey (NHANES) data from to 2007–2018. We hypothesized that a high CDAI could be associated with a reduced risk of albuminuria.

### Population studied

The NHANES is a long-term nationwide survey of the United States population that employs probability sampling techniques and complex multistage sampling methods. The goal of NHANES is to provide comprehensive data on the health and nutrition of the United States population. For more information on the continuous design of the NHANES, refer to http://www.cdc.gov/nchs/nhanes/index.htm. All research protocols were approved by the National Center for Health Statistics Ethics Review Board before data collection, and each participant provided informed consent.

**Figure 1:**
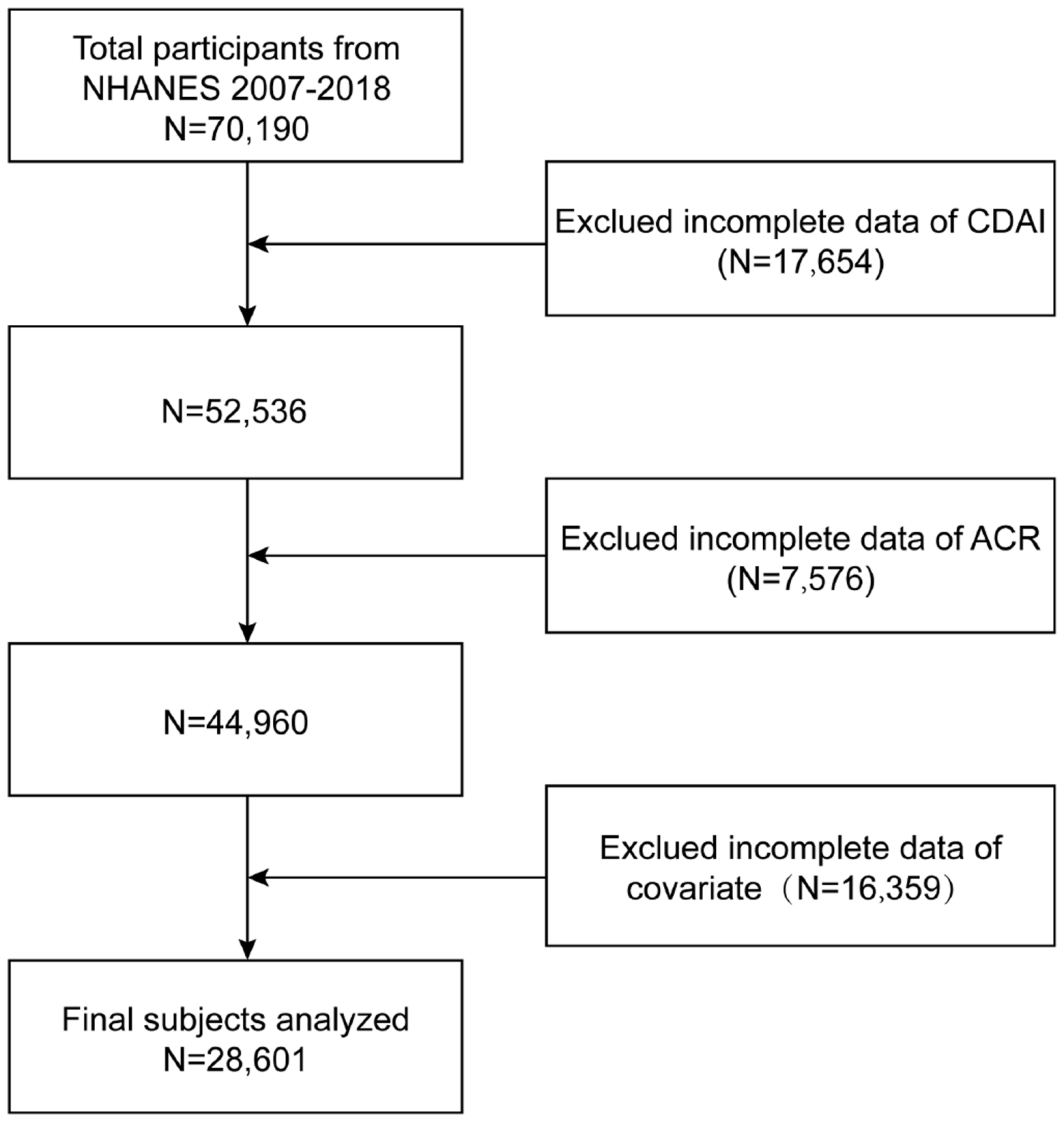
Flow diagram.

We excluded 17,654 participants with missing CDAI data, 7,576 participants with missing urinary albumin data, and 16,359 participants with missing relevant covariates from an eligible population of 70,190. After applying listwise deletion to participants with missing key data, the final study population included 28,601 individuals. The participant selection criteria are depicted in Figure 1.

### Evaluation of increased urinary albumin excretion

Urine samples were collected from NHANES participants at the mobile examination center. The levels of creatinine and albumin in the urine were measured using a modified Jaffe kinetic technique and solid-phase fluorescence immunoassays. The urine albumin content divided by the urine creatinine concentration yielded the ACR. Previous research has defined albuminuria, or excess urine albumin excretion, as an ACR > 30 mg/g (10, 11). Albuminuria was used as the outcome variable in this study.

### Assessment of CDAI

Professional census takers conducted two separate 24-h dietary recall interviews during the NHANES survey. Data collection and evaluation were based on the average of the two recalls. The initial recall was conducted in person at a mobile screening center, whereas the second interview was conducted over the phone 3–10 days later. Using food intake data from two non-consecutive days proved to be more reliable than single-day data. The participants were requested to recall specific meals and drinks consumed in the 24 h before the interview. We focused on six dietary antioxidants of interest: carotenoids *β*, zinc, selenium, vitamin A, vitamin C, and vitamin E. Antioxidants from dietary supplements, prescription drugs, or ordinary drinking water were not included in the estimates of dietary antioxidants. We used a modified version of a study conducted by Wright et al. to evaluate CDAI (12). Briefly, each dietary antioxidant intake was normalized by subtracting the mean and dividing by the standard deviation. Subsequently, standardized dietary antioxidant consumption was included to compute the CDAI (13).

### Covariates

The covariates of this study included demographic traits, lifestyle habits, health status, and laboratory tests, selected based on previous studies. Other biological factors that affected the current study included age, race, sex, marital status, drinking status, smoking status, body mass index (BMI), high-density lipoprotein cholesterol, triglycerides, ghrelin, glutamic oxaloacetic aminotransferase, glutamic alanine aminotransferase, albumin, glycosylated hemoglobin, serum calcium, estimated glomerular filtration rate (eGFR), coronary heart disease, hypertension, and diabetes mellitus.

### Statistical analysis

In this study, the CDAI was divided into four quartiles, ranging from Q1 to Q4. Categorical variables are reported as percentages, and continuous variables are expressed as means and standard deviations. For continuous variables, t-tests or chi-square tests were used to compare differences between participants categorized according to CDAI quartiles as well as between subjects with and without ACR (for categorical variables). We evaluated multivariate regression models using the NHANES complex sample design, employing three different models to investigate the relationship between the CDAI and ACR. Models 1, 2, and 3 represent unadjusted, partially adjusted, and completely adjusted models, respectively. To assess the stability of the findings, a sensitivity analysis was performed by converting the CDAI from a continuous variable to a categorical variable (quartiles). The odds ratio (OR) and 95% confidence interval (CI) were used to evaluate the relationship between CDAI and ACR. Subgroup analyses stratified by age, sex, eGFR, diabetes, hypertension, coronary heart disease, and BMI were performed to investigate variations in effect sizes among the populations. An interaction test was used to evaluate the heterogeneity of connections between subgroups based on this information. To verify the non-linear correlation between ACR and CDAI, we employed smoothed curve fitting techniques and generalized additive modeling (GAM). Both the general population and the sex subgroups were validated. A two-segment linear regression model was fitted to each interval and threshold effects were computed if a non-linear association was observed. A log-likelihood ratio test was run between a single-linear model and a two-segment linear regression model to determine whether a threshold exists. R Studio (version 4.2.2) and EmpowerStats (version 2.0) were used for statistical analyses. Statistical significance was set at *p* < 0.05.

## Results

### Baseline characteristics of participants

A total of 28,601 individuals with a mean age of 49.708 ± 17.618 years were enrolled, of whom 47.6% were male. The mean CDAI score was 0.302 ± 3.895. Urinary albumin excretion was high in 11.92% of participants. The CDAI was converted from a continuous variable to a categorical variable (quartiles). The likelihood of a high ACR declined as the CDAI quartiles increased (Table 1). For Q1, Q2, Q3, and Q4, the corresponding albuminuria risk was 13.89, 12.69, 10.99, and 10.11%, respectively. Significant differences (*p* < 0.05) were observed among the four CDAI quartiles in terms of age, race, sex, marital status, drinking and smoking habits, BMI, triglycerides, glutamic oxaloacetic and glutamic alanine aminotransferases, albumin, glycosylated hemoglobin, serum calcium, eGFR, ACR, hypertension, and diabetes mellitus. Conversely, young non-Hispanic White men with higher aspartate aminotransferase, alanine aminotransferase, albumin, total calcium, and eGFR and lower BMI and glycosylated hemoglobin were more likely to be in the highest CDAI quartile. Non-smokers and moderate drinkers had higher CDAI scores. Furthermore, participants without hypertension, diabetes, or coronary artery disease had higher Q4 scores. In addition, the general characteristics of participants stratified by ACR or non-ACR are detailed in Supplementary Table 1.

**Table 1:** Demographic characteristics based on CDAI quartiles.

CDAI	Q1(−7.08 – −2.1)	Q2(−2.19 – −0.41)	Q3(−0.41–1.88)	Q4(1.88–151.31)	*p*-value
	N = 7,150	N = 7,150	N = 7,150	N = 7,151	
Age, years	50.32 ± 17.89	50.29 ± 17.71	49.45 ± 17.62	48.77 ± 17.21	<0.001
BMI (kg/m^2^)	29.73 ± 7.17	29.60 ± 6.87	29.25 ± 6.78	28.73 ± 6.73	<0.001
Triglycerides (mg/dL)	147.76 ± 109.57	151.67 ± 110.24	157.24 ± 127.31	154.07 ± 122.10	0.004
HDL-C	53.34 ± 16.34	53.51 ± 15.95	52.88 ± 15.98	53.20 ± 16.07	0.072
ALT (U/L)	23.05 ± 20.01	24.33 ± 16.41	25.73 ± 24.51	26.70 ± 20.62	<0.001
AST (U/L)	24.61 ± 20.89	24.89 ± 12.80	25.52 ± 17.80	26.37 ± 16.58	<0.001
GHb (%)	5.81 ± 1.13	5.77 ± 1.08	5.73 ± 1.04	5.67 ± 0.99	<0.001
Albumin (g/dL)	4.17 ± 0.35	4.19 ± 0.35	4.23 ± 0.35	4.26 ± 0.36	<0.001
Total calcium (mg/dL)	9.39 ± 0.37	9.40 ± 0.37	9.40 ± 0.36	9.41 ± 0.36	<0.001
eGFR (ml/min/1.73 m^2^)	92.67 ± 25.40	93.01 ± 24.25	93.81 ± 23.57	94.48 ± 22.27	0.008
Gender, %					<0.001
Male	2,417 (33.80%)	3,050 (42.66%)	3,749 (52.43%)	4,397 (61.49%)	
Female	4,733 (66.20%)	4,100 (57.34%)	3,401 (47.57%)	2,754 (38.51%)	
Race, %					<0.001
Mexican American	1,121 (15.68%)	1,179 (16.49%)	1,117 (15.62%)	1,026 (14.35%)	
Other Hispanic	742 (10.38%)	690 (9.65%)	650 (9.09%)	607 (8.49%)	
Non-Hispanic White	2,878 (40.25%)	3,151 (44.07%)	3,304 (46.21%)	3,354 (46.90%)	
Non-Hispanic Black	1826 (25.54%)	1,477 (20.66%)	1,327 (18.56%)	1,300 (18.18%)	
Other Races	583 (8.15%)	653 (9.13%)	752 (10.52%)	864 (12.08%)	
Marital status, %					<0.001
Married	3,338 (46.69%)	3,733 (52.21%)	4,052 (56.67%)	4,023 (56.26%)	
Widowed	680 (9.51%)	607 (8.49%)	472 (6.60%)	407 (5.69%)	
Divorced	893 (12.49%)	821 (11.48%)	667 (9.33%)	722 (10.10%)	
Separated	298 (4.17%)	254 (3.55%)	201 (2.81%)	189 (2.64%)	
Never married	1,333 (18.64%)	1,163 (16.27%)	1,203 (16.83%)	1,244 (17.40%)	
Living with partner	608 (8.50%)	572 (8.00%)	555 (7.76%)	566 (7.91%)	
Smoking status, %					<0.001
No	3,735 (52.24%)	3,985 (55.73%)	4,047 (56.60%)	4,120 (57.61%)	
At least 100 cigarettes in life	3,415 (47.76%)	3,165 (44.27%)	3,103 (43.40%)	3,031 (42.39%)	
Alcohol consumption, %					<0.001
No	2,977 (41.64%)	2,541 (35.54%)	2,391 (33.44%)	2,257 (31.56%)	
1–3 cups per day	3,206 (44.84%)	3,678 (51.44%)	3,749 (52.43%)	3,829 (53.54%)	
More than 3 cups per day	967 (13.52%)	931 (13.02%)	1,010 (14.13%)	1,065 (14.89%)	
Diabetes, %					<0.001
No	5,956 (83.30%)	6,012 (84.08%)	6,131 (85.75%)	6,238 (87.23%)	
Yes	1,041 (14.56%)	988 (13.82%)	852 (11.92%)	721 (10.08%)	
Borderline	153 (2.14%)	150 (2.10%)	167 (2.34%)	192 (2.68%)	
Coronary heart disease, %					0.065
No	6,829 (95.51%)	6,840 (95.66%)	6,856 (95.89%)	6,890 (96.35%)	
Yes	321 (4.49%)	310 (4.34%)	294 (4.11%)	261 (3.65%)	
Hypertension, %					<0.001
No	4,369 (61.10%)	4,461 (62.39%)	4,727 (66.11%)	4,779 (66.83%)	
Yes	2,781 (38.90%)	2,689 (37.61%)	2,423 (33.89%)	2,372 (33.17%)	
Albuminuria, %					<0.001
No	6,157 (86.11%)	6,243 (87.31%)	6,364 (89.01%)	6,428 (89.89%)	
Yes	993 (13.89%)	907 (12.69%)	786 (10.99%)	723 (10.11%)	

HDL-C, high-density lipoprotein-cholesterol; ALT, alanine aminotransferase; AST, aspartate aminotransferase; glycohemoglobin; eGFR, estimated glomerular filtration rate.

### Association of CDAI with albuminuria

The findings of the multivariate regression analysis of CDAI and ACR risk are presented in Table 2. The analysis indicated a correlation between a higher CDAI and a lower incidence of ACR. Both Model 1 (OR = 0.96; 95% CI: 0.95, 0.97) and Model 2 (OR = 0.97; 95% CI: 0.96, 0.98) corroborated this inverse correlation. The effect sizes of ACR and CDAI remained negatively correlated; however, they decreased in Model 3 (OR = 0.99; 95% CI: 0.97, 1.00). The CDAI quartiles were used to conduct sensitivity analysis. Compared with quartile 1, participants in quartiles 2, 3, and 4 had progressively decreased risks of ACR (Q1: 1.00, Q2: 0.96, Q3: 0.89, and Q4: 0.88, respectively), with a *p*-value of 0.0101 for the trend test.

**Table 2:** Association of CDAI with albuminuria.

CDAI	Model 1		Model 2		Model 3	
	OR (95% CI)	*P*-value	OR (95% CI)	*P*-value	OR (95% CI)	*P*-value
Continuous	0.96 (0.95, 0.97)	<0.001	0.97 (0.96, 0.98)	<0.001	0.99 (0.97, 1.00)	0.0077
Categories						
Q1	Reference		Reference		Reference	
Q2	0.90 (0.82, 0.99)	0.0342	0.92 (0.83, 1.01)	0.0933	0.96 (0.87, 1.07)	0.4901
Q3	0.77 (0.69, 0.85)	<0.001	0.81 (0.73, 0.90)	<0.001	0.89 (0.79, 0.99)	0.0271
Q4	0.70 (0.63, 0.77)	<0.001	0.76 (0.68, 0.84)	<0.001	0.88 (0.78, 0.98)	0.0197
*P* for trend	<0.001		<0.001		0.0101	

Model 1: no covariates were adjusted.

Model 2: adjusted for sex, age, and race.

Model 3: adjusted for age, race, gender, marital status, drinking status, smoking status, body mass index (BMI), high-density lipoprotein-cholesterol, triglycerides, ghrelin, glutamic oxaloacetic aminotransferase, glutamic alanine aminotransferase, albumin, glycosylated hemoglobin, serum calcium, estimated glomerular filtration rate (eGFR), coronary heart disease, hypertension, diabetes mellitus.

OR, odds ratio.

95% CI, 95% confidence interval.

### Linear relationship between CDAI and urinary albumin

**Figure 2:**
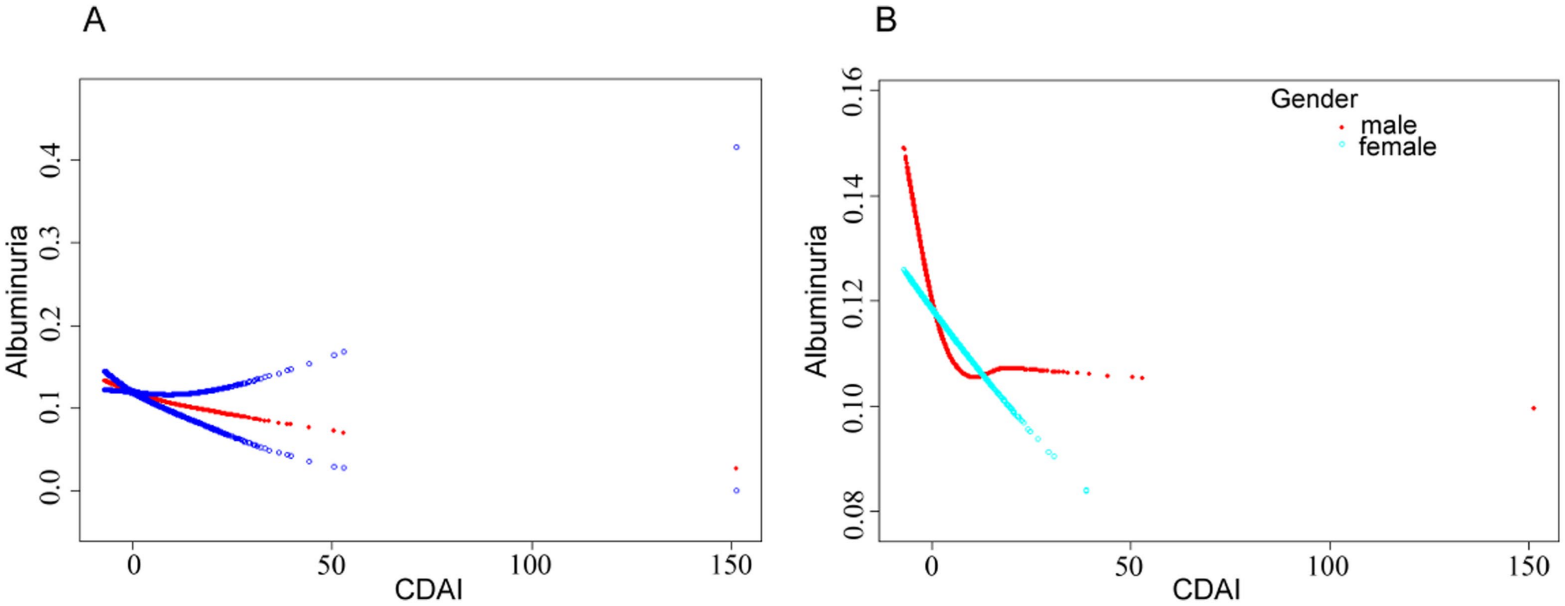
**(A)** Smoothed curve fit between CDAI and urinary albumin risk. **(B)** Smoothed curve fit between CDAI and urinary albumin risk after stratification by gender. The model adjusted for covariates such as age, race, sex, marital status, drinking status, smoking status, BMI, HDL-cholesterol, triglycerides, glutamic oxaloacetic transaminase, glutamic alanine aminotransferase, albumin, glycosylated hemoglobin, serum calcium, eGFR, coronary heart disease, hypertension, and diabetes mellitus.

The results revealed a linear association between urinary albumin level risk and CDAI by fitting a smooth curve (Figure 2A). An L-shaped curve association between CDAI and the risk of urinary albuminuria in male participants was discovered by further sex-stratified analysis (Figure 2B), with an inflection point of 0.53 (Table 3). On the left side of the inflection point, there was a negative correlation between CDAI and albuminuria (OR = 0.95, 95% CI: 0.91, 0.98), while there was no statistically significant correlation on the right side.

**Table 3:** Threshold effects after gender stratification.

		Male	Female	Total
Model 1	OR (95% CI)	0.99 (0.97, 1.00)	0.99 (0.97, 1.01)	0.99 (0.97, 1.00)
	*P* for trend	0.0626	0.2137	0.0077
Model 2	Breakpoint (K)	0.53	−4.4	0.73
	β1 (< K)	0.95 (0.91, 0.98)	0.84 (0.64, 1.10)	0.97 (0.95, 0.99)
	*P* for trend	0.0050	0.2053	0.0116
	β2 (>K)	1.00 (0.98, 1.02)	0.99 (0.98, 1.01)	0.99 (0.98, 1.01)
	*P* for trend	0.8118	0.3777	0.5186
	Logarithmic likelihood ratio test *p*-value	0.025	0.241	0.143

Model 1: Standard linear model.

Model 2: Two-piecewise linear model.

OR, Odds ratio.

95% CI, 95% confidence interval.

The total model was fully adjusted model; covariates other than gender were included in the adjustment after gender grouping.

### Subgroup analysis

**Figure 3:**
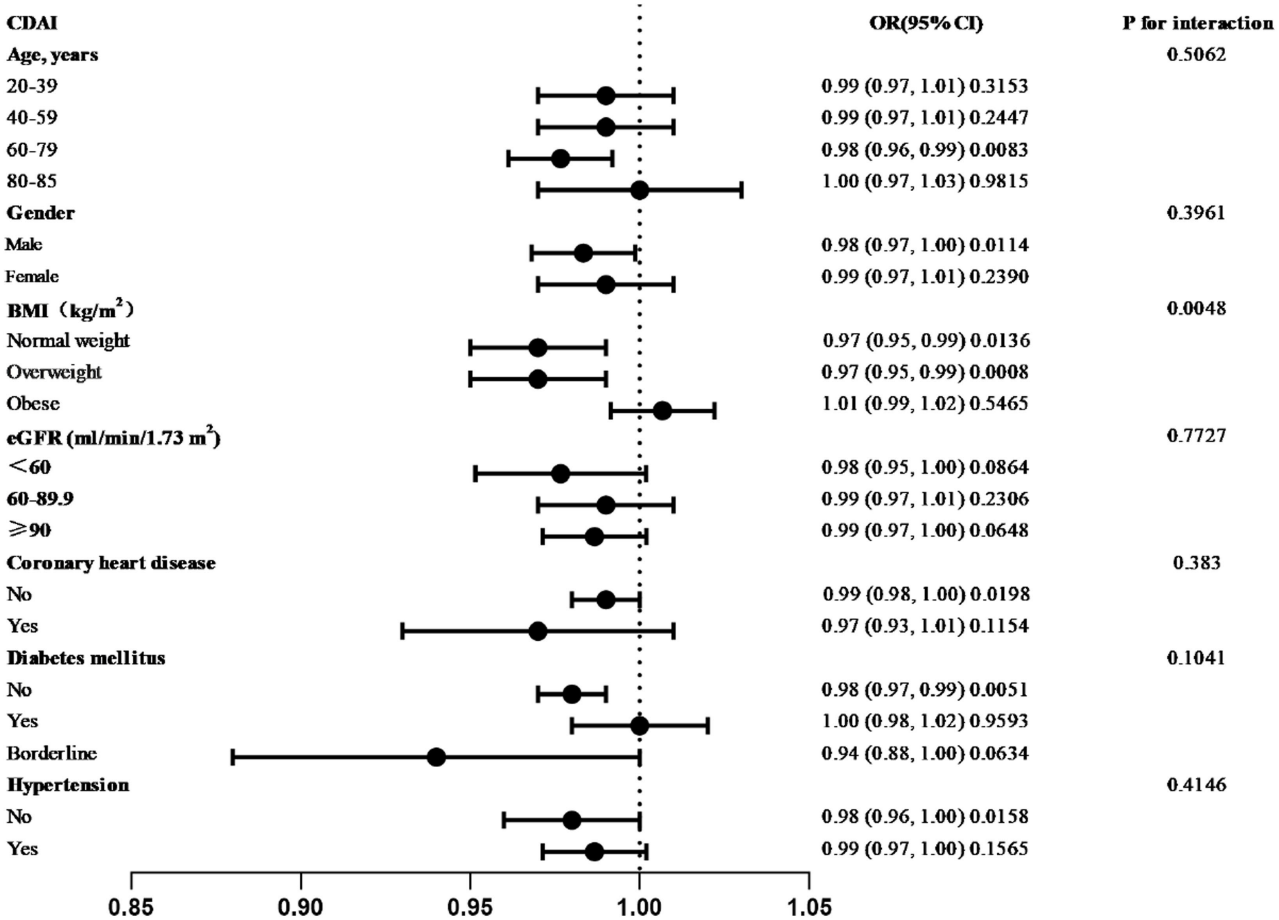
Forest plot of subgroup analysis between CDAI and urinary albumin.

Further subgroup analyses revealed inconsistent associations between CDAI and urine albumin levels (Figure 3). Participants aged 60–79, who were male, non-obese, with eGFR <60, had preclinical diabetes mellitus and coronary heart disease, and were not hypertensive exhibited a stronger correlation between CDAI and albuminuria in the subgroups by sex, age, BMI, eGFR, history of coronary heart disease, diabetes, and hypertension. Among these groups, only males who were neither obese nor hypertensive demonstrated a statistically significant difference (*p* < 0.05). The association between CDAI and urine albumin levels did not exhibit a significant dependency in the subgroups of sex, age, eGFR, coronary heart disease, diabetes, and hypertension according to an interaction test (*p*-value for interaction <0.05). However, we found a statistically significant difference (*p* < 0.05) in the BMI subgroups. These results suggest that BMI may alter the association between CDAI and albumin levels.

## Discussion

This study analyzed data from 28,601 adult United States residents who participated in the NHANES from 2007 to 2018. A correlation was observed between lower ACR and higher CDAI. The link between the two remained significant after adjusting for sex, age, and race in Model 2. This association was further maintained after incorporating all covariates in Model 3. A declining trend in ACR was anticipated as the CDAI increased according to the trend test versus the four CDAI categories (using Q1 as a reference; *p* = 0.01). These findings suggest that CDAI is associated with reduced oxidative stress and a lower likelihood of elevated ACR.

Previous studies have reported the relationship between CDAI and kidney disease, but the epidemiological approaches and clinical implications differ. Wang et al. (14) defined CKD by combining ACR and eGFR and investigated the associated with CDAI. Their study further revealed that CDAI intake prevented the development of CKD through multiple regression and subgroup analyses, emphasizing the importance of dietary interventions (14). However, a review suggested that glycocalyx activation in the endothelium reduces degradation and can lead to albuminuria and subsequent renal and vascular inflammation, thereby suggesting that albuminuria might bridge the progression of renal and cardiovascular diseases. Accordingly, this study highlights the important role of albuminuria in the progression of cardiovascular and renal diseases, and suggests that it should be used as an important indicator for clinical testing (15). Another cross-sectional study that included 13,584 participants reported a correlation between ACR and a human senescence suppressor gene (*α*-Klotho) and revealed a dose–response relationship between ACR and α-Klotho through GAM (16). Additionally, an experimental study involving albuminuria and cognitive impairment proposed that elevated albuminuria was associated with decreased hippocampal function and gray matter volume. Therefore, Bikbov et al. (17) concluded that albuminuria could be a risk factor for cognitive impairment. Based on these studies, we have strong reasons to believe that the clinical significance of ACR extends far beyond merely assessing kidney function and plays a crucial role in broader health contexts. Diets play an important role in regulating oxidative stress in the body and encompass the intake of various antioxidant-rich foods. Prior to the present study, many studies have used the CDAI as a marker to investigate the relationship between dietary antioxidants and health outcomes. Teng et al. (18) examined the relationship between CDAI and the incidence of stroke using the NHANES database, and found a strong negative correlation between CDAI and stroke risk. Based on this, a column chart model for the prediction of stroke was developed, and a ROC curve was further used to assess that the CDAI-based model had a strong negative correlation with stroke risk. The ROC curve assessed the good predictive efficacy of the model established based on CDAI (AUC = 77.4%) (18). Additionally, previous studies have explored the relationship between CDAI and other metabolic disorders, including hyperlipidemia (19), metabolic syndrome (20), and thyroid dysfunction (21). These conditions are associated with oxidative stress. A comprehensive review indicated that higher CDAI scores may be associated with albuminuria.

Current research has suggested a negative correlation between antioxidants and renal health, with biomarkers indicating associations with oxidative stress. This observation is consistent with our findings, further underscoring the pivotal role of antioxidants in the regulation of oxidative stress (14, 22). An elevated ACR is linked to podocyte damage, particularly affecting the slit diaphragm and actin cytoskeleton. Key genetic mutations in these structures have been identified, highlighting their role in the development of proteinuria (23–25). Oxidative stress causes further damage by increasing the production of ROS. This leads to lipid peroxidation, DNA damage, protein modifications, and activation of inflammatory pathways, resulting in renal cell apoptosis and worsening of kidney function (26, 27). Additionally, studies have reported that mice with compromised superoxide dismutase activity are prone to glomerulopathy and exhibit elevated ACR levels (28). However, research indicates that patients with albuminuria typically exhibit higher levels of oxidative stress and inflammation, and have relatively low intake of dietary antioxidants (29, 30), which further supports our conjecture.

Subgroup and interaction analyses were conducted. Notably, a significant difference was found between the categories only when the population’s BMI was divided into the normal, overweight, and obese groups (*p* = 0.0048). Analysis revealed a relationship between CDAI and ACR in the normal-weight and overweight groups, suggesting that higher antioxidant intake may lower ACR; however, this relationship was not observed in the obese group. Recent investigations have confirmed this association between obesity and ACR (10, 31, 32). One possible mechanism is that as BMI increases, the number of white adipocytes in the trunk also increases. These white adipocytes then release immuno-inflammatory factors, such as tumor necrosis factor-*α*, interleukin-6, interleukin-2, and c-reactive protein, gradually raising the body’s level of inflammation (33) and initiating an inflammatory cascade that damages the peduncle cells (34, 35).

The threshold analysis in this study revealed a linear relationship between CDAI and ACR in the overall population. However, in male participants, the relationship was characterized by an L-shaped curve. This relationship may be related to the differences in hormone production between men and women. The anti-inflammatory and antioxidant effects of estrogen in women may help them benefit from a wider range of antioxidants (36). Estrogen enhances the expression of antioxidant genes, thereby increasing antioxidant utilization and efficiency (37). This finding supports our observation that the dose–response relationship in women remains linear with increased antioxidant intake.

This relationship has been linked to differences in the body fat distribution between male and female participants, particularly the higher distribution of visceral fat in men (38, 39). These findings support the hypothesis that an increase in white adipocytes triggers an immune-inflammatory response, which plays a role in elevating ACR. When white adipocytes and lipocalin levels increased, the high ACR disappeared. Consequently, an L-shaped curve connection between CDAI and ACR may be obtained by judicious supplementation with antioxidant-rich foods to lower ACR (Figure 2).

This is the first study to suggest a relationship between CDAI and the likelihood of urinary albumin levels, which could have practical implications for adult healthcare in the United States. First, the NHANES provided a nationally representative dataset that serves as the basis for this study. The extensive and diversified sample size of the NHANES allows for improved statistical stability and precision, improving the generalizability of the findings to a wider population of adult U.S. citizens. Second, the CDAI, rather than specific antioxidants, may provide a more accurate indicator of total dietary antioxidant status than the exposure variables used in this study. However, this study has some limitations. Initially, efforts were made to consider the effect of covariates on outcomes, but we recognize that other potential confounders, such as medication use, existing subclinical nephropathy, and the use of dietary supplements other than those measuring vitamins and minerals, may also affect outcomes. Future studies should aim to incorporate these variables to better understand their impact on study outcomes. Second, we were only able to offer hints on the causal link between CDAI and albuminuria. Owing to the limitations of cross-sectional research, we were unable to elucidate this relationship. Therefore, it is necessary to conduct further prospective studies. In addition, due to the limitations of the database itself, some key data were missing, which triggered selective bias. Therefore, a more comprehensive program should be designed in the future to verify the robustness of the results. Finally, the self-reported approach may not accurately capture the true dietary habits of the participants, which may lead to biased antioxidant intake.

## Conclusion

This study revealed that increasing the CDAI is associated with a low ACR and reduced risk of albuminuria. This implies that national dietary guidelines, particularly the recommendations for patients with chronic kidney and cardiovascular diseases, could benefit from emphasizing antioxidant-rich diets to lower the risk of albuminuria, improve patient prognosis, and enhance overall survival.

## Data Availability

Publicly available datasets were analyzed in this study. This data can be found: https://www.cdc.gov/nchs/nhanes/index.html.
