# Supplementary material for: Association between composite dietary antioxidant index and increased urinary albumin excretion: a population-based study
Source: Front Nutr. 2025 Mar 28;12:1552889. doi: 10.3389/fnut.2025.1552889 (PMC11985420; doi:10.3389/fnut.2025.1552889)
Supplement: Supplementary file 1 [file Table_1.docx]

Supplementary table 1 General characteristics of participants stratified by ACR or non-ACR

|  | Non-ACR | ACR | *P*-value |
| --- | --- | --- | --- |
|  | N=25192 | N=3409 |  |
| **Age， years** | 48.46 ± 17.29 | 58.96 ± 17.25 | <0.001 |
| **BMI（kg/m^2^）** | 29.17 ± 6.79 | 30.46 ± 7.56 | <0.001 |
| **HDL-C** | 53.41 ± 15.93 | 51.91 ± 17.17 | <0.001 |
| **GHb (%)** | 5.65 ± 0.89 | 6.45 ± 1.75 | <0.001 |
| **Albumin (g/dL)** | 4.23 ± 0.35 | 4.11 ± 0.38 | <0.001 |
| **ALT(U/L)** | 24.94 ± 18.57 | 25.06 ± 31.99 | <0.001 |
| **AST(U/L)** | 25.18 ± 16.71 | 26.58 ± 20.95 | 0.12 |
| **Total calcium (mg/dL)** | 9.40 ± 0.36 | 9.41 ± 0.41 | 0.604 |
| **Triglycerides(mg/dL)** | 149.94 ± 114.20 | 173.03 ± 138.53 | <0.001 |
| **eGFR (ml/min/1.73 m^2^)** | 95.21 ± 22.42 | 80.84 ± 30.01 | <0.001 |
| **Gender, %** |  |  | 0.871 |
| Male | 11986 (47.58%) | 1627 (47.73%) |  |
| Female | 13206 (52.42%) | 1782 (52.27%) |  |
| **Race, %** |  |  | <0.001 |
| Mexican American | 3899 (15.48%) | 544 (15.96%) |  |
| Other Hispanic | 2393 (9.50%) | 296 (8.68%) |  |
| Non-Hispanic White | 11251 (44.66%) | 1436 (42.12%) |  |
| Non-Hispanic Black | 5098 (20.24%) | 832 (24.41%) |  |
| Other Races | 2551 (10.13%) | 301 (8.83%) |  |
| **[Marital status](C:/Users/Administrator/Desktop/环氧乙烷和脑卒中.docx#DMDMARTL), %** |  |  | <0.001 |
| Married | 13434 (53.33%) | 1712 (50.22%) |  |
| Widowed | 1654 (6.57%) | 512 (15.02%) |  |
| Divorced | 2669 (10.59%) | 434 (12.73%) |  |
| Separated | 804 (3.19%) | 138 (4.05%) |  |
| Never married | 4525 (17.96%) | 418 (12.26%) |  |
| Living with partner | 2106 (8.36%) | 195 (5.72%) |  |
| **Smoking status, %** |  |  | <0.001 |
| No | 14170 (56.25%) | 1717 (50.37%) |  |
| [At least 100 cigarettes in life](C:/Users/Administrator/Desktop/环氧乙烷和脑卒中.docx#SMQ020) | 11022 (43.75%) | 1692 (49.63%) |  |
| **Alcohol consumption, %** |  |  | <0.001 |
| No | 8587 (34.09%) | 1579 (46.32%) |  |
| 1-3 cups per day | 13027 (51.71%) | 1435 (42.09%) |  |
| More than 3 cups per day | 3578 (14.20%) | 395 (11.59%) |  |
| **Diabetes, %** |  |  | <0.001 |
| No | 22186 (88.07%) | 2151 (63.10%) |  |
| Yes | 2441 (9.69%) | 1161 (34.06%) |  |
| Borderline | 565 (2.24%) | 97 (2.85%) |  |
| **[coronary heart disease](C:/Users/Administrator/Desktop/环氧乙烷和脑卒中.docx#MCQ160c), %** |  |  | <0.001 |
| No | 24338 (96.61%) | 3077 (90.26%) |  |
| Yes | 854 (3.39%) | 332 (9.74%) |  |
| **Hypertension, %** |  |  | <0.001 |
| No | 16961 (67.33%) | 1375 (40.33%) |  |
| Yes | 8231 (32.67%) | 2034 (59.67%) |  |
